# Subnormal Growth Velocity and Related Factors During GnRH Analog Therapy for Idiopathic Central Precocious Puberty

**DOI:** 10.4274/jcrpe.0023

**Published:** 2018-07-31

**Authors:** Nursel Muratoğlu Şahin, Asiye Uğraş Dikmen, Semra Çetinkaya, Zehra Aycan

**Affiliations:** 1 University of Health Sciences, Dr. Sami Ulus Obstetrics and Gynecology, Pediatric Health and Disease Training and Research Hospital, Clinic of Pediatric Endocrinology, Ankara, Turkey; 2 Gazi University Faculty of Medicine, Department of Public Health, Ankara, Turkey

**Keywords:** Central precocious puberty, growth velocity, GnRHa therapy

## Abstract

**Objective:**

Data concerning subnormal growth velocity (GV) and factors that influence this during gonadotropin-releasing hormone analog (GnRHa) therapy for idiopathic central precocious puberty (ICPP) are scarce. We investigated the incidence of subnormal GV and associated factors in patients receiving GnRHa therapy for ICPP.

**Methods:**

In this retrospective cohort study, the records of 50 girls who had been diagnosed with ICPP and started on GnRHa treatment before the age of eight years were investigated. Subnormal GV frequency, related factors during GnRHa therapy and the effect on final height were examined.

**Results:**

During the treatment, a significant decrease in the annual GV and GV standard deviation score (SDS) of the patients was observed. In 16 (32%) patients GV never declined below -1 SDS, while a decline was noted once and twice in 19 (38%) and 15 (30%) patients respectively. The median age of detection of subnormal GV was 9.9 (4.9-10.9) years. Patients with pubic hair at diagnosis were found to have an increased risk of subnormal GV (p=0.016). There was a significant negative correlation between diagnostic basal luteinizing hormone (LH) level and the first and second year GV SDS (p=0.012 and 0.017 respectively). A significant negative correlation between bone age at diagnosis and 3^rd^ year GV SDS, and 4^th^ year GV SDS (p=0.002 and p=0.038) was also observed. LH suppression significantly increased during treatment (p=0.001).

**Conclusion:**

In girls with ICPP the risk of subnormal GV appears highest at the 3^rd^ year of GnRHa treatment, particularly in those patients with, at the time of diagnosis, pubic hair in conjunction with high baseline and peak LH and advanced BA and excessive LH suppression on follow-up.

## What is already known on this topic?

Data concerning subnormal growth velocity and factors that influence this during gonadotropin-releasing hormone analog therapy for idiopathic central precocious puberty are scarce.

## What this study adds?

In girls with idiopathic central precocious puberty the risk of subnormal growth velocity appears highest at the 3rd year of gonadotropin-releasing hormone analog treatment in those patients with, at the time of diagnosis, pubic hair in conjunction with high baseline and peak luteinizing hormone (LH) and advanced bone age and excessive LH suppression on follow-up.

## Introduction

Central precocious puberty (CPP) in girls is usually defined as the development of pubertal sex characteristics before the age of 8 years, usually as a consequence of the premature activation of the hypothalamic-pituitary-gonadal (HPG) axis. The pathogenesis of CPP includes early activation of pulsatile release of gonadotropin-releasing hormone (GnRH), leading to an increase in secretion of gonadotropins and gonadal steroids (1). In the majority of CPP cases, the etiology of the premature activation of the HPG axis is not clear. CPP in the absence of organic disease is known as idiopathic CPP (ICPP).

Children with precocious puberty tend to exhibit temporary accelerated growth due to increased sex hormones, but also a shorter growing period, which may ultimately lead to a shorter final height (FH). Early increased sex hormone concentrations shorten the growing period by promoting growth plate senescence, which refers to the structural and functional changes of the epiphyseal growth region including decline in chondrocyte proliferation and rate of longitudinal bone growth (2,3).

The mainstay of treatment for CPP is GnRH analogs (GnRHa). GnRHa bind to the GnRH receptors in the gonadotropic cells of anterior pituitary gland, resulting in desensitization of these receptors and eventual gonadal suppression due to down-regulation of the intrinsic pulsatile secretion of the LH and follicle-stimulating hormone (FSH) (4). This, in turn, reduces the growth velocity (GV), giving the long bones more time to lengthen before the growth plates fuse, thus increasing the FH that the child will achieve (5). However, in some patients, treatment with GnRHa does not just normalize GV but supresses it below the normal range (6,7,8,9). Previous studies have not revealed a clear hormonal cause for this phenomenon (10,11,12), raising the possibility that impaired growth during GnRHa therapy is due, at least in part, to premature growth plate senescence induced by prior estrogen exposure (13). Until now, during GnRHa treatment for ICPP, there is no cohort study for the evaluation of GV in which regular follow-up is performed from the beginning to the end of treatment. Therefore, during GnRH treatment the frequency of subnormal GV, time of occurrence, associated factors and the effect on FH are unclear.

In this retrospective cohort study, for the patients who had GnRHa treatment with ICPP diagnosis, GV records obtained at three month periods were investigated from the beginning until the end of treatment. The rate and time of occurrence of subnormal GV, factors associated with subnormal GV, and the effect on FH were investigated.

## Methods

The records of 50 girls who had a diagnosis of ICPP in our clinic between July 2010 and July 2013, who had been started on GnRHa treatment before the age of eight years and who had completed the treatment with regular follow-up were investigated. Since the study was retrospective, ethics committee approval and informed consent were not taken. 

All subjects experienced breast development, Tanner stage B2, as a first sign of puberty before eight years of age and all were premenarcheal at presentation. The girls were diagnosed with ICPP if chronological age (CA) at onset of breast development was <8 years and peak luteinizing hormone (LH) level was more than 5 IU/L (5 mIU/mL) in response to 2.5 µg/kg (maximum 0.1 mg) GnRH (0.1 mg Gonadorelin acetate, Ferring^®^) and if brain magnetic resonance imaging was normal. All subjects had recently experienced rapid growth in height and/or one or more than one year advance in bone age (BA) assessed by the method of Greulich and Pyle. Target height was calculated by mid parental height minus 6.5 cm. Target height range was calculated by target height ±5 cm. Leuprolide acetate 1-month depot was started as 3.75 mg/dose administered intramuscularly every 28 days, at the time of ICPP diagnosis.

Follow-up assessments were performed every three months. Follow-up study visits included a physical examination with measurement of height and weight, assessment of Tanner stage. Determination of LH levels at 30 and 60 minutes after the GnRHa injection were performed every 6 months. Height and GV standard deviation score (SDS) were determined using anthropometric reference data for Turkish children (14). GV was considered subnormal if the GV was below -1 SDS. Patients with suboptimal pubertal suppression (clinical pubertal progression and peak LH response to the GnRHa >3.3 U/L) were excluded from the study. Treatment was discontinued in patients who had a CA of 11 or who had a CA of 10.5-11 and in conjunction with a BA of 12 years. 

After drug withdrawal, visits were continued every six months until menarche, and then annually until the patient reached FH. Girls were considered to be at FH if they were growing less than 0.5 cm/year or if BA was greater than or equal to age 16 years. 

Exclusion criteria included CPP caused by organic lesions, being born small for gestational age, thyroid disease, intake of any other medications, presence of chronic diseases or growth-affecting medical problems. A drop-out case was defined as one who did not complete the follow-up described in the protocol, which includes voluntary discontinuation of treatment, irregularity of visits, treatment incompatibility, side effects of treatment, detection of additional diseases that may affect growth and suboptimal pubertal suppression. 

Serum FSH and LH levels were measured by immunofluorometric assays (ARCHITECH System, Abbott Laboratory Diagnostics, USA) with detection limits of 0.05 mIU/mL and 0.07 mIU/mL for FSH and LH, respectively. The intraassay and interassay CV was 3.2% in both gonadotropin assays. 

### Statistical Analysis

The data were entered into the SPSS 21.0 computer package program and analyzed. Qualitative data are presented as numbers/percentages, while quantitative data are given as means, medians and standard deviations. Nonparametric tests were used after the normal distribution conformity test. For the comparison of two groups Mann-Whitney U test was used and for three groups’ comparison the Kruskal-Wallis test, where p<0.05 was considered statistically significant.

## Results

A total of data on 808 follow-up visits of 50 female patients who had received ICPP treatment before age eight years and had been evaluated at regular check-up visits, were evaluated. The median follow-up period was 48±10.5 (33-72) months and 31 patients reached FH. The clinical, laboratory and radiological findings of patients are given in Table 1. Twenty-four patients were observed to have a FH compatible with midparental height (MPH) (MPH ±5 cm). The FH of five patients was above MPH, and only two patients were below MPH.

**Figure 1:**
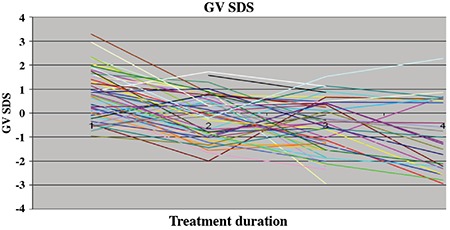
The annual growth velocity standard deviation score of patients GV: growth velocity, SDS: standard deviation score

During the treatment period, a significant decrease in the annual GV and GV SDS of the patients was observed (p=0.02 and p=0.001 respectively) (see Table 2 and Figure 1). Although GV of patients did not decline below -1 SDS in the first year of treatment, GV dropped below -1 SDS in 28.2% (11 of 39) in the second year, 41.7% (20 of 48) in the third year, 50% (13 of 26) in the fourth year, 33.3% (2 of 6) in the 5^th^ year and in 75% (3 of 4) in the 6^th^ year of treatment. The median age of detection of subnormal GV was 9.9 (4.9-10.9) years. The median time of subnormal GV occurrence was the 3^rd^ year of treatment (minimum-maximum 2-6 years). In the third and fourth years of treatment, CA of patients who showed a subnormal GV was 10.12±0.67 and 10.28±0.77, respectively. The CA of patients who showed a subnormal GV was higher than that of patients who showed a normal GV (p=0.005 and p=0.045 respectively) (Table 3). During the treatment period GV never declined below -1 SDS in 16 (32%) of the patients, while in 19 patients’ (38%) a decline below -1 SDS was noted once and in 15 patients (30%) twice. GV of patients who attained a FH below MPH never dropped below -1 SDS.

It was found that basal LH levels of patients (0.46±0.69) who had at least one subnormal GV episode were higher than the basal LH levels of patients (0.12±0.08) who did not have subnormal GV episodes (p=0.044). It was found that peak LH levels of patients (mean 8.82±3.63) who showed two episodes of subnormal GV were considerably higher than the peak LH levels of patients (mean 6.59±3.43) who showed only one subnormal GV episode (p=0.039).

When the patients were stratified according to age of diagnosis, as either between 3.0-6.9 years old group (group 1) or 7.0-8.0 years old (group 2), it was seen that group 2 had a lower 3^rd^ and 4^th^ year GV SDS compared to group 1 (p=0.0001, p=0.011 respectively). However, there was no difference in the frequency of declining of GV below -1 SDS.

When the relationship between initial diagnostic parameters and the annual GV SDS was examined (Table 4), it was found that the age of diagnosis was significantly negatively correlated with the 3^rd^ year GV SDS, and 4^th^ year GV SDS (p=0.0001 and p=0.009 respectively). There was also a significant negative correlation between height at diagnosis and 3^rd^ year GV SDS (p=0.019). There was a significant positive correlation only between basal FSH level at diagnosis and 3^rd^ year GV SDS (p=0.046). There was a significant negative correlation between basal LH level at diagnosis and GV SDS in the first and second years (p=0.012 and p=0.017 respectively). Basal estradiol at diagnosis was significantly negatively correlated with first year and 4^th^ year GV SDS (p=0.020 and p=0.028). It was found that there was a significant negative correlation between the BA at diagnosis and the 3^rd^ and 4^th^ year GV SDS (p=0.002 and p=0.038). Advanced BA (BA-CA) at diagnosis was found to be negatively correlated only with first year GV SDS (p=0.005). There was a significant positive correlation between MPH and 2^nd^ year GV SDS and 3^rd^ year GV SDS (p=0.014 and p=0.041), but no correlation was found between GV SDS, FH and FH-MPH. There was a significant positive correlation between the duration of treatment and 3^rd^ and 4^th^ year GV SDS (p=0.001 and p=0.008).

There was no correlation between breast stage at diagnosis and GV SDS. In addition, the incidence of subnormal GV was not different according to the breast stage at diagnosis. There was no correlation between the pubic hair stage at diagnosis and GV SDS. When the relation between pubic hair stage at diagnosis and subnormal GV was evaluated, in 85.7% (n=18) of patients with no pubic hair no subnormal GV SDS occurred, whereas there was at least one episode of subnormal GV in 82.4% (n=14) of the patients with pubic hair. Patients with pubic hair at diagnosis were found to have an increased risk of subnormal GV (p=0.016).

The LH suppression level of all patients was evaluated in the first two years of treatment. This evaluation could be performed in only 47 patients in the third year of treatment, 26 patients in the fourth year of treatment and six patients in the fifth year of treatment. It was observed that LH suppression increased significantly over the treatment years (p=0.001). The second year LH of the patients who showed a subnormal GV in the 3^rd^ year of treatment was significantly more suppressed (mean=0.87±0.37 minimum-maximum: 0.49-1.70) than those with normal GV values (mean=1.20±0.53 minimum-maximum: 0.40-2.83) (p=0.030), although no significant correlation was found between LH suppression level and GV SDS.

## Discussion

Although some studies have reported subnormal GV in some GnRHa-treated patients with ICPP, factors associated with subnormal GV were not investigated in these studies (6,7,8,9) or only one year of treatment was evaluated (13). Our study is the first cohort study to investigate the frequency, time of occurrence, and factors associated with subnormal GV and its effect on FH during GnRHa treatment in patients with ICPP.

In this study, the GV and GV SDS significantly decreased over the years of GnRHa treatment. Although the GV SDS was within the normal limits during the first year of treatment, GV began to decline below -1 SDS, starting from the second year and during the treatment interval and in 58% of the patients, GV dropped below -1 SDS at least once. The cause of linear growth impairment during GnRHa treatment is unknown. Several investigators have examined the effect of gonadal suppression with GnRHa on the growth hormone axis and height velocity. Although some studies have suggested a subnormal GH secretion during treatment with GnRHa (11,15), others have not (10,12,16). Evaluation of subpopulations of children with poor growth during GnRHa therapy has also not clearly demonstrated GH deficiency (8,11,15,17,18,19). Furthermore, studies have reported no significant change in IGF-1 and IGFBP-3 concentrations, despite a decrease in the height velocity (20,12). Lack of change in IGF-1 and IGFBP-3 with decrease in sex hormones level and height velocity suggests a direct effect of sex hormones on growth. *In vitro* and animal studies have shown that sex steroids may act via locally produced IGF-1 in the target tissues without significantly raising circulating IGF-1 concentrations (4). According to Weise et al (13), height velocity SDS is correlated inversely with markers of the severity of prior estrogen exposure, including duration of precocious puberty before treatment start and Tanner breast stage. However, no correlation between estradiol and GV was found in this study (13). It is known that activation of the hypothalamus-pituitary gonad (HHG) axis in puberty results in LH dominant secretion rather than FSH in the LHRH test, thus stimulating estrogen production in the ovaries. When we examined the relationship between GV and the HHG axis there was a positive correlation between basal FSH at diagnosis and 3^th^ year GV SDS, a negative correlation between basal LH at diagnosis and 1^st^ and 2^nd^ year GV SDS and a negative correlation between estradiol and first year and 4^th^ year GV SDS values. Patients whose GV was subnormal at least once were found to have higher basal LH levels at diagnosis than LH levels of patients with normal GV. Peak LH levels of patients who showed subnormal GV twice were higher than the peak LH levels of patients who had one subnormal GV episode. These findings support the hypothesis that, during GHRHa treatment, there was an increased risk of subnormal GV as the degree of activation of the HHG axis increases. However, there was no correlation between the breast stage at diagnosis and GV SDS. In normal pubertal development, pubic hair growth also starts not long after the onset of breast development. In our study, an increased risk of subnormal GV in patients with pubic hair at diagnosis also suggests that the initiation of treatment in the later stages of puberty may increase the risk of GV decrease. Weise et al (13) have shown that height velocity SDS in the second year of treatment is correlated inversely with BA advancement and that BA was the best independent predictor of growth during GnRHa therapy. These authors hypothesized that during GnRHa therapy, when hormonal concentrations are normalized, this excessive senescence would be expected to result in decreased linear growth. Similar to these results, in our study we also found that BA at diagnosis showed a significantly negative correlation with the third and the fourth year GV SDS. Advanced BA (BA-CA) at diagnosis was found to be negatively correlated only with the 1 year GV SDS. In the study of Weise et al, (13) it was suggested that subnormal GV was related to the fact that 40% of their patients were postmenarcheal, to late onset treatment (maximum 9.4 years) and to an advanced BA (maximum BA 14 years, median BA advance 3.8 years). Of note in our study a correlation was found between BA and GV, despite the fact that all of our patients were premenarcheal, the treatment was initiated before the age of eight years and BA was not greatly advanced (maximum BA 12 years, mean BA advance 1.36 years). One of the best indicators of estrogenic effect in precocious puberty is advanced BA. In addition, the finding that advanced CA at diagnosis is correlated with GV and GV SDS only in the 1^st^ year of treatment suggests that the effect of the removal of estrogenic activity by GnRHa treatment on GV SDS is only present at the beginning of the treatment. In our study, while advanced BA at diagnosis had no effect on GV SDS after the first year of treatment, BA at diagnosis appeared to be related to GV SDS at the 3^rd^ and 4^th^ years. These results suggest that subnormal GV SDS after the first year of treatment was independent of advance in BA and associated with only with the level of bone maturation. In our study, the negative correlation between age of diagnosis and 3^rd^ and 4^th^ year GV SDS also supports this conclusion.

It has also been hypothesized that GnRHa treatment might inhibit growth by suppression of estrogen concentrations to levels below those of prepubertal children (21). However, estrogen measurements using an ultrasensitive recombinant cell bioassay are not consistent with this hypothesis (22). Possible excessive suppression of estradiol could not be demonstrated due to the fact that the estradiol kit used in our study was not ultrasensitive. However, the significant increase in LH suppression with GnRHa treatment over the years and having more suppressed LH levels at the second year of patients with subnormal GV at 3 years of treatment were considered to be a significant effect of LH suppression on GV. However, considering the absence of a relationship between treatment dose and LH suppression, it was concluded that the degree of LH suppression varied independently from the dose and that excessive suppression of LH should be avoided in the management of these patients.

The effect of subnormal GV seen in patients receiving GnRHa therapy with ICPP on FH is not known exactly. There are studies which report that for some patients treated with GnRHa, GV decreases so considerably that patients fail to reach their target height (23,17). In our study, positive correlations between MPH and 2^nd^ and 3^rd^ year GV SDS showed that LH suppression with GnRHa in precocious puberty provided appropriate growth in accordance with the patient’s genetic potential. Subnormal GV was frequently observed in patients treated with GnRHa treatment, but the FH of the patients was compatible with MPH. FH and MPH-FH were not different in patients with subnormal GV and normal GV. 

In our study, we found that age of diagnosis was negatively correlated with 3^rd^ and 4^th^ year GV SDS. A subnormal GV was observed in the 3^rd^ year of treatment (median) and the median age of detection of subnormal GV was 9.9 (4.9-10.9) years. It was seen that patients who started treatment at 7-8 years had a lower GV and GV SDS than patients who started treatment at a younger age. Thus, it was thought that patients, who started treatment at age 7-8 years might be at risk of subnormal GV when they are 10-10.5 years old.

### Study Limitations

The two limitations in our study were, firstly, a possible excessive suppression of estradiol could not be demonstrated due to the fact that the estradiol kit used in our study was not ultrasensitive. Secondly, FH and MPH-FH were not different in patients with subnormal GV and normal GV in our study. The effect of subnormal GV on FH may have been small due to occurrence of subnormal GV that is often observed at the end of treatment. New studies are needed to investigate the effect of the subnormal GV seen in the first years of treatment on FH, since the subnormal GV in our study was observed in the last years of treatment in many subjects.

## Conclusion

This study shows that that during GnRHa treatment in ICPP, the risk of subnormal GV is high in the 3^rd^ year of treatment and/or at ages 10-10.5 years, in patients with pubic hair at diagnosis, in those in whom the treatment is started at ages 7-8 years, in those who have a high baseline level and peak LH at diagnosis and in those with advanced BA and excessive LH suppression on follow-up. Subnormal GV during GnRHa treatment did not affect FH.

## Figures and Tables

**Table 1:**
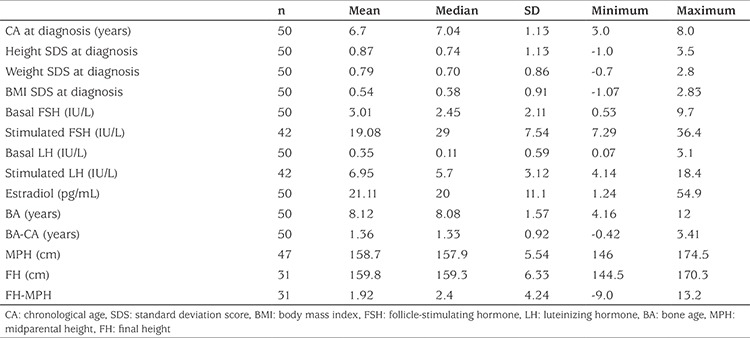
The clinical, laboratory and radiological findings of the patients

**Table 2:**
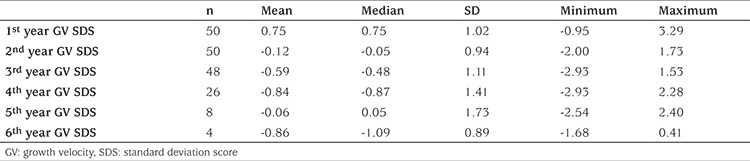
The annual growth velocity standard deviation score of the patients

**Table 3:**
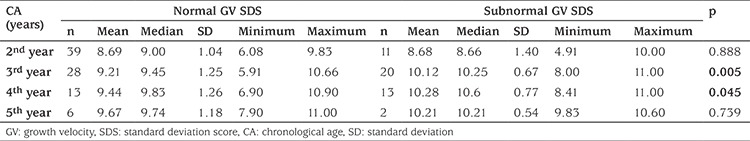
Chronological age of patients who had normal and subnormal growth velocity standard deviation score by year

**Table 4:**
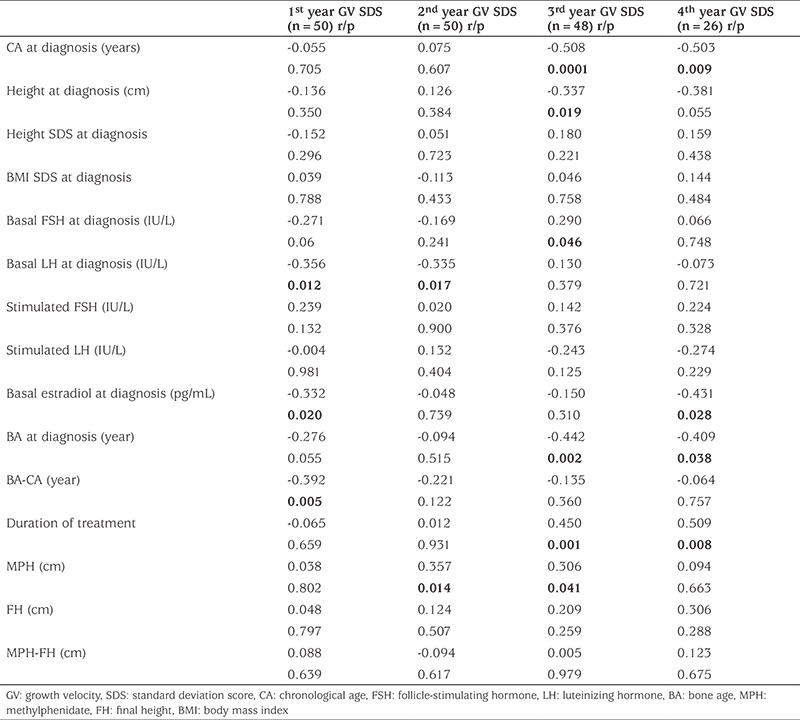
The relationship between annual growth velocity standard deviation score and clinical features
